# Vaccines against Tuberculosis: Where Are We Now?

**DOI:** 10.3390/vaccines11051013

**Published:** 2023-05-22

**Authors:** Shruti Srivastava, Sajal Dey, Sangita Mukhopadhyay

**Affiliations:** 1 Research and Development Office, Ashoka University, Rajiv Gandhi Education City, Sonipat 131029, Haryana, India; shruti.shrivastava@ashoka.edu.in; 2 Laboratory of Molecular Cell Biology, Centre for DNA Fingerprinting and Diagnostics (CDFD), Inner Ring Road, Uppal, Hyderabad 500039, Telangana, India; sajaldey@cdfd.org.in; 3 Graduate Studies, Manipal Academy of Higher Education, Manipal 576104, Karnataka, India

**Keywords:** tuberculosis, *Mycobacterium tuberculosis* (*M.tb*), *Bacille Calmette–Guérin* (BCG), vaccines, immune responses, clinical trial, public health

## Abstract

Tuberculosis (TB) is among the top 10 leading causes of death in low-income countries. Statistically, TB kills more than 30,000 people each week and leads to more deaths than any other infectious disease, such as acquired immunodeficiency syndrome (AIDS) and malaria. TB treatment is largely dependent on BCG vaccination and impacted by the inefficacy of drugs, absence of advanced vaccines, misdiagnosis improper treatment, and social stigma. The BCG vaccine provides partial effectiveness in demographically distinct populations and the prevalence of multidrug-resistant (MDR) and extensively drug-resistant (XDR) TB incidences demands the design of novel TB vaccines. Various strategies have been employed to design vaccines against TB, such as: (a) The protein subunit vaccine; (b) The viral vector vaccine; (c) The inactivation of whole-cell vaccine, using related mycobacteria, (d) Recombinant BCG (rBCG) expressing *Mycobacterium tuberculosis* (*M.tb*) protein or some non-essential gene deleted BCG. There are, approximately, 19 vaccine candidates in different phases of clinical trials. In this article, we review the development of TB vaccines, their status and potential in the treatment of TB. Heterologous immune responses generated by advanced vaccines will contribute to long-lasting immunity and might protect us from both drug-sensitive and drug-resistant TB. Therefore, advanced vaccine candidates need to be identified and developed to boost the human immune system against TB.

## 1. Introduction

Tuberculosis (TB) is a major health problem in low-income countries. It is estimated that 10.6 million new active TB cases and 1.6 million TB deaths occur annually [1]. TB accounts for the highest number of deaths caused by a single infectious disease surpassing AIDS (acquired immunodeficiency syndrome) and malaria; the COVID-19 pandemic is an exception here. The discovery of vaccine-mediated protection was revolutionary in the history of modern medicine and helped to save millions of lives [2]. Despite this, the only tuberculosis vaccine that has been available for over a century is *Bacille Calmette–Guérin* (BCG) [1,3]. Infection with *M.tb* bacterium in humans causes pulmonary (lung) tuberculosis (pTB). Infected individuals often do not show symptoms of infection immediately; *M.tb* stays silently in the body and re-activates later to manifest TB symptoms. TB can spread to other parts of the body, such as bones, the brain, and stomach, and can lead to mortality. Treating TB is largely dependent on BCG vaccination and anti-TB drugs [1,4].

**Figure 1:**
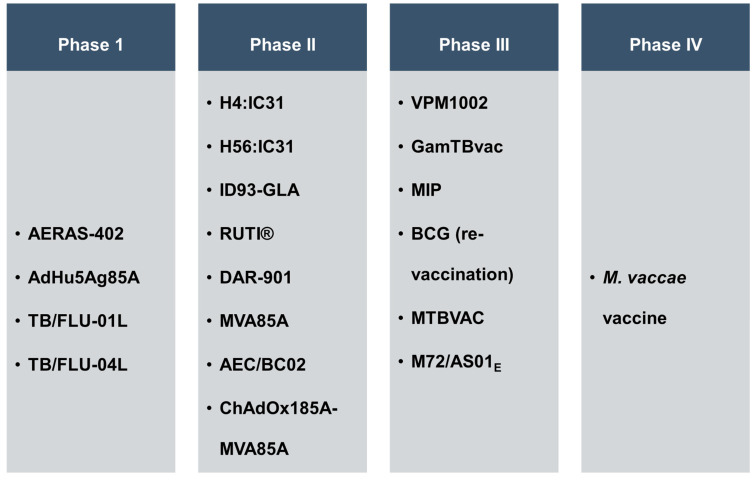
Clinical trial status of vaccine candidates against tuberculosis.

The BCG vaccine is a live, weakened form of *M. bovis*, given to infants via intradermal (ID) route. BCG vaccination is a preventive approach; however, protection against TB is often lost by adolescence or early adulthood and fails to prevent pulmonary tuberculosis. This results in the re-activation or dissemination of TB infection. Several studies on the BCG vaccine have shown that BCG vaccination lacks the ability to develop varied, diversified and distinct cell-mediated immune responses in humans, which could eradicate *M.tb* infection [3,5]. Infection with drug-resistant *M.tb* strains is another challenge and poses a great threat to public health as it has a very poor cure rate and high cost of treatment. TB treatment is further impacted by the co-infection of HIV (human immunodeficiency virus). Statistically, one-third of the 34 million humans infected with HIV are co-infected with *M.tb*, and 25% of HIV-related deaths are due to TB [1]. Thus, there is an urgent need to develop new vaccines that will generate trained as well as heterologous, long-lasting immune responses and protect against *M.tb* infection. In recent years, there have been major advancements in the area of vaccine development, many of which are in various phases of clinical trials and include many strategies have been introduced to design vaccines against TB (Table 1, Figure 1) [4,6,7], such as: (a) The protein subunit vaccine; (b) The viral vector vaccine; (c) The inactivation of whole-cell-based vaccine using related mycobacteria; (d) The rBCG vaccine,. Figure 1 reveals that a good numbers of TB vaccine candidates are in Phase II and Phase III of clinical trials, which is a healthy sign for the TB vaccine development strategy.

## 2. Description of Various Vaccine Candidates Designed against TB

### 2.1. Fusion Protein Candidates

#### 2.1.1. M72/AS01_E_

The M72/AS01_E_ vaccine is being developed by GlaxoSmithKline (GSK). M72/AS01_E_ contains two *M.tb* antigens, *M.tb*32A and *M.tb*39A, in combination with AS01_E_ adjuvant [8,9]. The vaccine generated antigen-specific polyfunctional T cell populations after vaccination in BCG-vaccinated, HIV-negative adults infected with *M.tb* versus adults not infected with *M.tb* [34]. Prolonged secretion of M72-specific antibodies and CD4^+^ T cells was observed in M72/AS01_E_-vaccinated participants, and pulmonary TB were found to be lower in these participants [34]. M72/AS01_E_ induced cellular and humoral immune responses in HIV patients [9]. M72/AS01_E_ is in Phase III clinical trials [4].

#### 2.1.2. H4:IC31

In H4 fusion protein, *M.tb* antigens, Ag85B, and TB10.4 are combined together along with an adjuvant IC31. Chemically, IC31 is a combination of leucine-rich peptide (KLK) and a synthetic oligonucleotide (ODN1a). H4:IC31 is under investigation as a booster vaccine to enhance BCG efficacy [10]. Intramuscular administration of H4:IC31 in BCG-vaccinated adults could generate antigen-specific, long-lasting, and strong CD4^+^ T cells. H4 and IC31 together induce antigen presentation, interferon-gamma (IFN-γ) secretion, and T cell effector immune responses [10,35]. H4:IC31 is in Phase II clinical trials [4].

#### 2.1.3. H56:IC31

In the H56 fusion protein, *M.tb* antigens, Ag85B, ESTA-6, and Rv2660) are combined with an adjuvant, IC31. This vaccine is being developed by Statens Serum Institute (Copenhagen, Denmark) in partnership with Valneva (Lyon, France), GmBH (Vienna, Austria), Aeras, Rockville, MD, USA, the South African TB Vaccine Initiative (SATVI) Cape Town, South Africa, The European, and Developing Countries Clinical Trials Partnership (EDCTP), Research Council of Norway and Valneva, International AIDS Vaccine Initiative (IAVI) New York, NY, USA. A clinical trial by Luabeya et al. (2015) showed that vaccination with H56:IC31 could trigger a heterogenous population of effector CD4^+^ T cells in HIV-negative, BCG-vaccinated *M.tb*-uninfected adults; additionally, in *M.tb*-infected adults, monofunctional IFN-γ-expressing antigen-specific CD4^+^ T cells were observed [11]. H56:IC31 is promising for its ability to prevent TB in BCG-vaccinated, HIV-negative adolescent individuals. Vaccination with H56:IC31 generated strong CD4^+^, as well as H56:IC31-specific antibodies in the study participants [35]. H56:IC31 is in Phase II clinical trials [4].

#### 2.1.4. ID93-GLA

The ID93 subunit vaccine is a fusion polyprotein and consists of *M.tb* antigens Rv2608, Rv3619c, Rv3620c, Rv1813c. These *M.tb* antigens have been found to be upregulated in *M.tb*-exposed individuals. Glucopyranosyl lipid adjuvant (GLA) is a Th1-inducing synthetic Toll-like receptor (TLR) 4-agonist. When combined, the ID93-GLA vaccine could prevent the growth of *M.tb* in mice and guinea pigs in prophylactic application. Furthermore, ID93 was tested for its therapeutic application in mice and non-human primates [36]. The ID93 vaccine is under investigation for two purposes; (1) Prevention of disease in BCG-vaccinated, *M.tb*-uninfected or infected HIV-negative individuals; (2) Its therapeutic potential in the prevention of *M.tb* infection in HIV-negative or HIV-positive individuals who had received treatment for drug-sensitive TB in the past [12,36]. ID93 vaccine is in Phase II clinical trials [4].

#### 2.1.5. GamTBvac

GamTBvac is a recombinant protein subunit vaccine, consisting of two *M.tb,* Ag85A and ESAT-6/CFP-10. They are combined together with a dextran binding domain from *Leuconostoc mesenteroides*), immobilized on dextran and mixed with an adjuvant. The adjuvant has a DEAE-dextran core and CpG oligodeoxynucletides. Phase II clinical trials in BCG-vaccinated, *M.tb*-uninfected adults showed that the GamTBvac vaccination produced had strong immunogenicity, polyfunctional CD4^+^ T cells, and increased the secretion of IFN-γ. Vaccine-specific IgG antibodies were also observed, indicating the humoral immune response [13]. GamTBvac is in Phase III clinical trials [4].

#### 2.1.6. AEC/BC02 

AEC/BC02 is a combination of *M.tb* protein subunits Ag85A and ESAT-6/CFP-10(EC) and an adjuvant. The adjuvant has BCG-derived unmethylated cytosine–phosphate–guanine (CpG) and aluminum salt [14]. Previous studies have demonstrated that BC02 can increase antigen-specific interleukin-12 (IL-12) response by peritoneal macrophages and induce stronger T-helper (Th) 1 and Th2 responses [37]. The AEC/BC02 vaccine induces a Th1-mediated cellular immune response against the reactivation of latent *M.tb* infection in mice after chemotherapy [14]. AEC/BC02 is in Phase II clinical trials [38].

#### 2.1.7. Recombinant BCG (rBCG) Vaccine

*M.tb* and BCG share 98% of their genome. In principle, the identification of *M.tb*-specific antigens and designing a TB subunit vaccine with BCG will not only bolster the efficiency of the BCG vaccine but also induce antigen-specific trained immune responses [39]. Designing a *M.tb*-specific vaccine with multiple antigens that do not cross-react with BCG is dependent on the following parameters: (i) Not being immunogenic in the context of BCG vaccination; (ii) Having previously reported immunogenicity in *M.tb*-infected humans; (iii) The induction of protective immune response in animal models. Based on this, new BCG vaccines are being developed either by: (a) Overexpressing the immunogenic antigens of *M.tb* on the backbone of BCG; or (b) Knocking out certain immunogenic BCG antigens and using these rBCG strains as carrieriers to express *M.tb* antigens. *M.tb* antigens on the backbone of BCG could stimulate T cell cross-presentation functions and boost the efficacy of the BCG vaccine. A fusion protein (CMX) composed of immunodominant epitopes from Ag85C, MPT51, and HspX was expressed in BCG to generate a recombinant BCG (rBCG). This induced specific Th17 and Th1 cells against TB infection in mice [40]. Additionally, Deng et al. (2014) used a recombinant *Bacille Calmette–Guérin* expressing Ag85A-ESAT-6 (rBCG-AE). The rBCG-AE vaccination increased Th1 cellular immune responses against *M.tb* infection in mice [41]. Gengenbacher et al. (2016) showed that deleting nuoG from *M. bovis* BCG ΔureC::hly imparts better protection against tuberculosis in the mice model [42]. Indeed, the efforts to improve the potential for rBCG vaccines to elicit protective humoral responses were also undertaken by various groups [43,44,45], but none of these approaches were very successful. Thus, there is still a demand to generate a novel genetically engineered BCG vaccine with enhanced immunogenicity (T cell and B cell) that can protect against *M.tb* infection, as compared to the existing BCG vaccine. BCG revaccination is another option in the TB vaccine development. When the BCG vaccine was re-administered into BCG-vaccinated individuals, it showed improved CD4^+^ T-cell responses post BCG revaccination [16]. BCG revaccination is in Phase III clinical trials [4].

The secretory proteins of *M.tb* perform a diverse set of functions, such as metabolism of mycobacteria, modulating host immune responses, and these proteins impart adaptability and virulence to *M.tb* [44,46,47]. Some of these proteins in the mycobacteria prevent phagosome maturation and acidification, and decrease antigen presentation. Therefore, rBCG with the deletion of those secretory genes or expressing *M.tb*-specific secretory proteins are being tested as vaccine candidates [38,39]. AFRO-1 is an rBCG overexpressing *M.tb* antigens Ag85A, Ag85B, TB10.4 and perfringolysin O from *Clostridium perfringens*. Perfringolysin O helps the antigens to escape into the cytoplasm. C57BL/6 mice vaccinated with rBCG (AFRO-1) showed enhanced immune response and survival against *M.tb* (strain HN878) infection, as compared to BCG control. Interestingly, AFRO-1 was also safe to use in severe combined immunodeficiency (SCID) mice [15]. Pym et al. (2003) showed that mice and guinea pigs vaccinated with rBCG expressing ESAT-6 were able to secrete ESAT-6 and protect against *M.tb* infection [48]. A separate research study by Horwitz et al. (2000) showed that a rBCG vaccine expressing the *M.tb* 30-kDa major secretory protein could induce greater protective immunity against *M.tb* than the conventional BCG vaccine [49].

The *M.tb* antigen 85 (Ag85) complex is a family of immunodominant and 30–32 kDa secretory proteins (Ag85A, Ag85B, and Ag85C) and has been included in several vaccine designs [50,51,52]. Ag85C (fbpC, Rv0129c) is singularly important for producing approximately 40% of the total mycolic acid in *M.tb* [52]. Mycolic acid is an important constituent of the cell wall biosynthesis pathway. The expression of Ag85C has been shown to be upregulated in *M.tb-*infected macrophages [48,50]. Thus, Ag85C appears to be a good candidate for creating the rBCG vaccine. A fusion protein vaccine that consists of Ag85B, the 190-198 peptide of Mpt64, HspX (Rv2031c), and *M.tb* 8.4 (Rv1174c) has been shown to enhance BCG-mediated immunity and provide better protection against *M.tb* infection [53]. A rBCG expressing secretory fusion protein containing *M.tb*-Ag-85B + murine IL-15 (rBCG-Ag85B-IL15) was capable of inducing efficient cell-mediated immunity and induced robust protection against *M.tb* challenge, as compared to BCG alone [51]. Again, α-crystallin (acr, HspX, Rv2031c) is a member of dormancy regulon and highly upregulated in *M.tb* in response to hypoxia, scarcity of nutrition or during infection. α-crystallin is another good target for generating recombinant BCG vaccine [54,55]. A DNA vaccine based on α-crystallin was shown to activate Th1 response and reduce bacterial load in guinea pigs when infected with *M.tb*. The rBCG vaccine overexpressing α-crystallin was able to strengthen the protective function of BCG [56,57,58,59,60,61,62,63,64,65,66,67]. Another strategy was used where a rBCG strain overexpressing antigen HspX (rBCG::X) was constructed. Immunization with rBCG::X in mice showed lower bacterial load in lung and less severe lung pathology than the control mice vaccinated with BCG strain. rBCG::X could provide better protection in mice against infection with *M.tb* [54]. As, Ag85- and α-crystallin-based rBCG vaccine candidates have induced reduction in the bacillary load in the lungs and spleen, as well as decreased histopathological damage, and an increased and sustained protection in animals compared to BCG [50,51,52,53,54,55,56]; these rBCG vaccines are crucial in the TB vaccine design.

Importantly, the tuberculosis vaccine clinical trial expert group (TVCTEG) under the Department of Biotechnology, Government of India, have recommended several rBCG vaccines for conducting human clinical trials, which are: (i) rBCG overexpressing antigen 85C; (ii) rBCG overexpressing a-crystallin as the priming agent followed by boosting with a DNA vaccine expressing the same antigen, α-crystallin; (iii) BCG as priming agent followed by boosting with DNA vaccine expressing α–crystallin [52,53,54,55,56]. All these studies focus on improving the function of existing BCG vaccines, which have some practical advantages as the protocol; infrastructure for the BCG vaccination program is already in place and introduction of these simple, new, and improved BCG vaccines may result in better clinical management of TB patients [57].

#### 2.1.8. VPM1002

In the VPM1002 vaccine candidate, a rBCG Prague strain expresses listeriolysin of *Listeria monocytogenes*. In this strain, the listeriolysin molecule from *Listeria* has inactivated urease subunit C. Listeriolysin helps the antigen to escape from the macrophages [58]. Grode et al. (2005) reported that rBCG equipped with listeriolysin of *Listeria monocytogenes* showed the secretion of mycobacterial antigens in the extracellular milieu, which are accessible to the antigen-presenting cells of the immune system. This reduced the bacterial load and provided protection against *M.tb* infection in mice [59]. VPM1002 vaccination is safe, and it generated an antigen-specific CD4^+^ and CD8^+^ T cell response [18,58]. A clinical trial of the VPM1002 vaccine candidate is underway to evaluate its efficacy in the prevention of recurrence of pulmonary or extrapulmonary TB infection in HIV-negative adults [4], as well as in HIV-exposed and HIV-unexposed newborns [4]. VPM1002 is in Phase III clinical trials.

#### 2.1.9. BCG + H107

Vaccine H107 contains eight *M.tb* antigens: Rv 3873, ESAT-6, EspI, EspC, EspA, MPT64, MPT70, and MPT83. These antigens are either absent or have negligible expression/secretion in the BCG strain. The administration of H107 along with BCG improved adaptive responses against *M.tb* infection [17]. Importantly, co-administration of H107 and BCG stimulated the development of BCG-primed T cells, as well as new T cell clonal populations against *M.tb* antigens in mice [17]. As a result, T cells exhibit subdued Th17 responses and less differentiated Th1 cells. The *M.tb*-specific subunit vaccine H107 can also be administered alone [17].

### 2.2. Viral-Mediated Delivery of Mycobacterial Antigen

Virus or viral particle-based vectors are small, efficient and have excellent transfer potential. These viral vectors are genetically modified or engineered to overexpress antigens and generate immune responses. Thus, they are used as a delivery agent in gene therapy. In TB, vaccinia virus, adenovirus and influenza virus vectors are the most investigated as viral-vectors. However, the immunogenicity of viral vectors may affect vaccine performance or cause adverse reactions.

#### 2.2.1. MVA85A

MVA85A is a modified, replication-deficient vaccinia virus-based vaccine, expressing *M.tb* antigen 85A (Ag85A). MVA85A was found to be safe and well tolerated in BCG-vaccinated adults and induced robust cellular immune responses [19,20]. Phase I clinical studies on MVA85A were promising and MVA85A was found to be safe and immunogenic in healthy adults infected with HIV [60,61], but when Phase clinical trials was conducted, it did not offer significant protection against *M.tb* infection in BCG-vaccinated infants. However, MVA85A was safe and well-tolerated when administered as a booster to BCG vaccine [62]. Tameris et al. (2013) have reported that in response to MVA85A vaccination, the CD4^+^ T cells were produced but CD8^+^ T cells were absent or significantly low [62]. Thus the immunogenicity of MVA85A was weak and ineffective to prevent *M.tb* infection in HIV-negative infants. One of the arguments was that, as the immune system of infants are not fully developed, they failed to generate robust immune responses post MVA85A vaccination [62]. Though, MVA85A may have some advantages, it requires a rigorous pre-clinical and clinical studies to plan and include it into the vaccine development pipeline [60,61,62,63].

#### 2.2.2. Adenovirus Based Vaccine Candidate

Adenoviruses stimulate robust innate and adaptive immune responses owing to their strong immunogenicity. In humans, more than 50 serotypes of adenoviruses are known but adenovirus type 5 (Ad5) is the most studied viral vector [64]. A recombinant, replication-deficient human Ad5 vector expressing *M.tb* antigen, Ag85A (AdHu5Ag85A) is in Phase I clinical trials [4]. Although immunization with Ad5Ag85A were able to activate antigen specific CD4^+^ and CD8^+^ T cells in mice [21,22], intranasal administration, but not intramuscular administration of vaccine generated long-lived CD8^+^ T-cells in the airway lumen suggesting that the route of vaccine administration (intranasally or intramuscular) could determine the distinct immune profiles [21]. Ad5Ag85A vaccine has been studied in different animal models, such as calves, guinea pigs, goats using various routes of vaccine administration, such as intramuscular, intradermal or endobronchial. The vaccine was reported to be safe and able to protect against various strains of mycobacteria (*M. bovis*, *M.tb*, *M. caprae*) [22,65,66,67,68].

AERAS-402 is based on replication-deficient adenovirus, Ad35 and contains *M.tb* antigens (85A, 85B, and TB10.4). These *M.tb* antigens are potent T cell epitopes and immunogenic. Phase I clinical trials showed that AERAS-402 was safe and no serious adverse effects were observed among HIV-negative and BCG-vaccinated adults. Immune profiling studies indicated induction of a robust polyfunctional CD8^+^ T cell response and secretion of pro-inflammatory cytokines such as, IFN-γ, tumor necrosis factor-alpha (TNF-α) and IL-2 [23]. Another study indicated that AERAS-402 is safe in healthy infants previously vaccinated with BCG [69]. Though Ad5 is an important viral vector for consideration in TB vaccine, however, natural immunity against Ad5 limits the use of Ad5 as a viral delivery vector. To overcome this problem, an alternate strategy was developed to use rare serotype of adenovirus or non-human primate-derived adenovirus or employ different route of vaccine administration [70].

#### 2.2.3. TB/FLU-01L

TB/FLU-01L is a live vector vaccine consisting of replication-deficient influenza virus A expressing ESAT-6 antigen. It is developed by the Research Institute of Influenza (Saint Petersburg, Russia). A Phase clinical trial of the TB/FLU-01L tuberculosis vaccine when administered intranasally or sublingual in BCG-vaccinated healthy adults revealed that the vaccine is safe and well-tolerated. In this study, no adverse reaction against viral carrier particles was observed, and 70% of the vaccinated subjects could respond to *M.tb* antigens [24].

#### 2.2.4. TB/FLU-04L

TB/FLU-04L is another viral vector vaccine based on the replication-deficient influenza virus expressing two *M.tb* antigens, Ag85A and ESAT-6 [6]. Preclinical studies in mice showed induction of strong T helper 1 (Th1)-type immune response and reduced regulatory T cells (Treg cell), when administered as booster to BCG [71]. It is in Phase I clinical trials [4].

#### 2.2.5. ChAdOx185A-MVA85A

ChAdOx185A-MVA85A is based on a replication-deficient chimpanzee adenovirus vector expressing *M.tb* antigen 85A (Ag85A). BCG-ChAdOx185A-MVA85A was safe and protective compared to BCG control when mice were immunized with it in the preclinical studies [72]. The addition of ChAdOx185A to BCG or MVA85A, was well-tolerated and safe to use in adults [5]. Another Phase I clinical trial has been conducted to investigate whether the immunogenicity of the vaccine depends on the routes of vaccine administration, i.e., aerosol versus intramuscular (IM) in healthy adult humans. Aerosol administration of vaccine ChAdOx1-85A has induced higher mucosal cellular responses, especially the IFN-γ/IL-17^+^ CD4^+^ T cells and IM vaccination produced higher systemic cellular and humoral responses [73]. This report opens a new area of research on the identification of the optimum route of vaccine administration. If the knowledge on how the stages and routes of vaccine administration can affect immune responses is available, it can help us to plan TB treatments and management with accuracy [5,72,73]. It is in Phase II clinical trials [4].

#### 2.2.6. RhCMV/TB

Researchers have developed a recombinant vaccine RhCMV/TB, a rhesus cytomegalovirus vector expressing *M.tb* antigens. When rhesus macaques were infected with *M.tb* (Erdman strain) 1 year post-vaccination, vaccinated macaques had stronger effector-differentiated CD4^+^ and CD8^+^ memory T cell responses against *M.tb* antigens compared to unvaccinated control and diminished *M.tb* infection. The results have been exceptional in explaining how immune responses can be trained to provide long-term protection with vaccines [25].

#### 2.2.7. rLCMV-Based *M.tb* Vaccine

The replication-deficient lymphocytic choriomeningitis virus (rLCMV)-based vaccine vector expressing *M.tb* antigens Ag85B and TB10.4 (Ag85B-TB10.4) was tested in neonatal and adult mice. It was observed that after administration, this vaccine was capable of generating heterogeneous CD4^+^, CD8^+^ T cell populations, and lung damage was reduced when infected with *M.tb* [26]. rLCMV may be developed as a novel vector for delivering mycobacterial antigens.

### 2.3. M.tb Auxotrophs, Mutants, Whole Cells or Heat-Inactivated Fragments as Vaccine Candidates

#### 2.3.1. MTBVAC

MTBVAC is a live attenuated vaccine that was developed from the *M.tb* clinical strain Mt103, thus retaining its ability to induce strong immune responses. Two virulent genes, *phoP* and *fadD26*, have been deleted from this vaccine candidate [74]. Preclinical studies showed that the MTBVAC vaccine was highly immunogenic and as safe as BCG when challenged with *M.tb* infection in mice and guinea pigs [75]. Stronger CD4^+^ T cell-dependent responses were observed in healthy adults (HIV-negative and BCG-vaccinated) immunized with MTBVAC [76]. In clinical trials, it is used as a preventive vaccine in newborns [27]. MTBVAC is a safe and promising candidate vaccine that is undergoing Phase III clinical trials to determine its ability to protect infants exposed to HIV from tuberculosis in areas of Sub-Saharan Africa where tuberculosis is endemic [4].

#### 2.3.2. DAR-901

DAR-901 is an inactivated strain of non-tuberculous mycobacteria, *Mycobacterium obuense*, prepared from the master cell bank of agar-grown strain, SRL172. Encouraging results were obtained in Phase I clinical trials, and currently, the Phase II trial is in progress [4,77]. DAR-901 is a BCG booster vaccine that can be used in TB-endemic areas. Additionally, it has been investigated for its safety and immunogenicity in HIV-infected adults against TB infection [77].

#### 2.3.3. IKEPLUS

IKEPLUS is a genetically modified *M. smegmatis* from which the ESX3 locus was deleted and complemented with a *M.tb* ESX-3 type VII secretion system (*M. smegmatis* Δesx-3 locus (IKE)+ *M.tb* esx-3 genes) [78]. The IKEPLUS strain was able to stimulate CD4^+^-dependent mycobactericidal immunity [29]. The IKEPLUS strain may serve as a vector candidate for the delivery of mycobacterial agents [29] and produce a distinct immunological and cytokine profile against *M.tb* infection [79]. This vaccine candidate is not in clinical trials.

#### 2.3.4. *M. vaccae* Vaccine

Heat-inactivated non-tuberculous bacterium *M. vaccae* is another vaccine candidate for the prevention of *M.tb* infection in TB-endemic areas or high-risk groups. In Phase III clinical trials, this vaccine was given as a tablet to drug-resistant and drug-sensitive TB patients; improved bacterial clearance and reduction in anti-TB drug-related toxicity was observed. It has shown a good therapeutic potential as an adjunct therapy to TB treatment [30]. Another *M. vaccae*-based vaccine, known as “*Mycobacterium Vaccae* for Injection (Mica)”, is in Phase IV clinical trials to assess the potential of “Mica” in preventing the development of TB in patients with latent TB infection [80]. Mica is being developed by Anhui Zhifei Longcom Biologic Pharmacy Co., Ltd. Beijing, China [80].

#### 2.3.5. RUTI^®^

The RUTI^®^ is being developed by Archivel Farma, S.L. Barcelona, Spain. The RUTI^®^ vaccine is a fragments of heat-inactivated *M.tb* in liposome suspension. Since RUTI^®^ has multiple *M.tb* antigens, it can induce strong adaptive immune responses. The RUTI^®^ vaccine showed that it was capable of generating a polyfunctional T cell response, and enhanced the secretion of IFN-γ in HIV-infected individuals in response to latent TB infection [31]. It is in Phase II clinical trials to assess its therapeutic potential in reducing the course of anti-TB drug treatment in multidrug-resistant and sensitive TB infection [4].

#### 2.3.6. *Mycobacterium indicus pranii* (MIP)

Immuvac, or *Mycobacterium indicus pranii*, displays antigens similar to both the leprosy bacterium and the TB bacterium. It was originally developed to prevent leprosy but currently it ability to prevent pulmonary tuberculosis is being investigated, and is in Phase III clinic trials [4,32].

## 3. Newer Avenues in TB Vaccine Research

### 3.1. Multiepitope DNA-Based Dual Vaccine for HIV and TB

*M.tb* antigens MPT64, Ag85A, Ag85B, and TB10.4 were incorporated onto the backbone of an HIV protein, p24. The resultant multiepitope DNA-based dual vaccine was immunogenic and elicited a T cell-mediated response in vitro, but in vivo vaccinated mice developed mild lung inflammation, limiting its utility [33]. Due to the negative results, this vaccine did not enter clinical trial; however, this study put forth a newer concept of developing a dual vaccine for HIV–TB co-infection [81,82].

### 3.2. Aptamers-Based TB Vaccine

In principle, aptamers are simple oligonucleotide molecules conjugated with various agents, such as siRNA, nanoparticles, and drugs [83,84]. Aptamers have recently gained the interest of the scientific community in diagnostic and therapeutic applications in cancer, anemia, age-related macular degeneration (AMD), influenza, and TB [83]. An aptamer ZXL1 has been shown to bind specifically with *M.tb*-ManLAM. ZXL1 was able to lower CD11^+^ expression in dendritic cells infected with *M.tb*, and prevented *M.tb* replication in mice and rhesus monkeys [85]. ZXL1 is a promising vaccine candidate that can differentiate between BCG and *M.tb* and impart protection from TB. The aptamer BM2 increases the immunogenicity of BCG against *M.tb* and H_37_Rv infection in monkeys and in mice [86]. The BM2 aptamer targets the BCG–ManLAM–CD44 interaction, and initiates M1 macrophage-mediated Th1-type T cell immune responses [86]. *M.tb*-Apt1 and *M.tb*-Apt6 are single-strand DNA (ssDNA) aptamers that target the activity of the *M.tb*-Acetohydroxy acid synthase (ASHS) enzyme. ASHS is important for the synthesis of essential amino acids in *M.tb* and has no known analogues in human to date. ASHS converts pyruvate molecules to acetoacetate. *M.tb*-Apt1/*M.tb*-Apt6 could suppress the ASHS function, resulting in killing *M.tb* bacilli and preventing infection [87]. Aptamer-based vaccine development is an emerging concept and has the potential to develop advanced vaccines and aid in drug development against TB, provided it receives systematic research in this area [83,84,85,86,87].

### 3.3. RNA/DNA and Peptide-Based Vaccines

RNA/DNA-based vaccines are rapidly evolving concepts in the vaccine development plan. It was attempted in an in silico, immunoinformatics study to design a multi-epitope mRNA vaccine that will have strong immunogenicity [88]. Mycobacterial MPT83 is an immunodominant antigen. MPT83 RNA- or MPT83 DNA-vaccinated mice showed a MPT83-specific CD8^+^ T cell response. MPT83 DNA-vaccinated mice displayed longer protection as compared to MPT83 RNA vaccination [89]. The potential of RNA/DNA-based vaccines in the treatment of infectious disease is yet to be fully explored and has the potential of providing a framework for vaccine design in the future [90]. The immunogenicity of the peptide vaccine is lower and unable to provide long-term protection. Immunodominant small peptides are recognized by antigen-presenting cells, and trigger a specific immune response. This lays the foundation for designing small, antigenic peptides recognized by the immune cells. Peptides based on the Ag85 complex (Ag85A or Ag85B), α-crystallin, and Rv1733c are some of the most notable examples for vaccine design [91]. Another such type is LT70, consisting of ESAT6-Ag85B-MPT64(190-198)-Mtb8.4-Rv2626c [91]. There are also approaches to consider peptide-based vaccines derived from PE/PPE family proteins, and a recent study indicates novel peptide-based vaccines named MP3RT and ACP [91].

### 3.4. PE/PPE Proteins as Vaccine Candidates

The PE/PPE family proteins are named after the repetitive occurrences of proline-glutamate (PE) and proline–proline–glutamate (PPE), respectively, having a conserved region at N-terminus. PE/PPE family proteins are predominantly present in the pathogenic *M.tb* and *M.tb* complex (MTBC) species with the sporadic occurrence of PE/PPE proteins being reported in non-pathogenic or environmental *Mycobacterium* sp. Strains; thus, they are thought to play an important role in host–pathogen interactions and the virulence of *M.tb*. The PE/PPE family represents nearly 10% of the *M.tb* genome, and several reports have shown that PE/PPE proteins help in subverting the host’s immune responses to establish successful colonization in the host [92,93]. PE/PPE proteins possess multiple antigenic epitopes and variations leading to the development of various sub-domains within the PE/PPE family, e.g., PE-PGRS, PPE-PPW, PPE-MPTR [47,94]. An interesting study by Delogu and Brennan (2001) has indicated that PE_PGRS33 can produce a prophylactic effect in mice as both a DNA and protein subunit against *M.tb* infection [94]. The PE4 (Rv0160c) protein has been reported to have approximately 19 antigenic epitopes and is immunogenic. Mice, when immunized with PE4, showed higher TNF-α levels, a lower bacterial load, and better survival upon *M.tb* challenge compared to BCG control [95]. Additionally, it was observed that PE4 protein could induce the generation of Th1/Th2 cytokine profiles [95]. Another PE/PPE family protein, PPE57, is a cell-wall-associated protein of *M.tb*, and produces a TLR2-dependent Th1 response. When mice were exposed to *M.tb* infection, the bacterial load was reduced in rBCG-PPE57-immunized mice compared to BCG exposure alone (96). Both PE4 and PPE57 are considered potential vaccine candidates against *M.tb* infection [95,96]. The immunization of mice with recombinant PE3 protein could induce a robust protective immune response against a challenge by live mycobacteria, and could also be considered as a subunit vaccine candidate [97]. Another antigen, PPE14 (MTB41), improved protection against *M.tb* in both mouse and guinea pig models [98]. Protein PPE15 induced significant protection when administered in the mice model to boost BCG vaccination [99]. The use of a polyprotein subunit vaccine (fusion of MPT65 with PE domain of PE_PGRS33) resulted in the increased efficacy of the BCG vaccine against *M.tb* infection in mice [100]. Importantly, three PPE proteins, PPE18 (Rv1196), PPE42 (Rv2608), and PPE68 (Rv 3873), are TB vaccines that are in clinical trials (Table 1).

### 3.5. HIV–TB Co-Infection and Application of Vaccines

Loss of immune function is a common and characteristic feature of both HIV and TB infections. HIV-infected people are at 18–20% higher risk of developing TB [1,101], and it contributes to 8% of total TB cases worldwide. In 2020, roughly 214,000 people living with HIV had succumbed to TB. HIV–TB co-infection is rampant in African countries; nearly 50% of the entire HIV–TB co-infection cases are reported from this region [101].

In general, there are two scenarios of TB infection in HIV-positive individuals: (i) By reactivation; or (ii) By acquired or de novo infection [101,102]. In HIV–TB patients, the adaptive immune response is severely compromised. HIV infection causes steep reduction in CD4^+^ T cells, which negatively affects CD4^+^/CD8^+^ T cell balance, rendering the host inefficient in controlling secondary infections, which limits the options of designing vaccines to manage HIV–TB co-infection. Macrophage infected with *M.tb* have been shown to favor HIV replication [103]. The clinical management of HIV–TB infection is marred for several reasons. Not only is TB diagnosis difficult in HIV-infected individuals, but the drugs used as anti-retroviral therapy (ART) and anti-TB treatment interfere with each other [104]. The regular use of these drugs causes adverse reactions, liver and digestive complications, mental fatigue, stoppage of drug courses; all these factors negatively impact the management of HIV and TB, and dependency on drugs needs to be reduced in HIV–TB co-infection [104,105]. Vaccine development should focus on developing vaccines that can be used in HIV–TB co-infection; this could be a joint HIV–TB vaccine or a stand-alone TB vaccine that is effective and safe to use in HIV–TB patients. Currently, MVA85A [61], M72/AS01_E_ [8,9], VPM1002 [4,18], RUTI^®^ [31], ID93-GLA [4,12,36], and DAR-901 [4,77] vaccines have been considered for clinical trial in the management of TB and HIV infection.

## 4. Challenges in TB Vaccine Research and Innovations—The Tip of the Iceberg

The vaccine development pipeline is extensive, intensive, and expensive. However, vaccine development faces several scientific and non-scientific challenges that hinder its progress. Some of the obstacles in the vaccine development path will be discussed in this section of the review. After investing billions of dollars into the research ecosystem, we have a decent, if not excellent, research infrastructure, standardized protocols, clinical trial networks, collaborative interest among various stakeholders, i.e., scientists, researchers, medical practitioners, and healthcare workers. To meet the demands of vaccination against TB, detailed basic research is required to identify virulence factors, delivery modes, adjuvants, as well as clinical and translational approaches.

### 4.1. Intracellular Behaviour of M.tb Is Unclear

*M.tb* possesses many virulent factors that interact with host cells and interfere with the host’s immune responses. However, the ways in which *M.tb* survives in the hostile environment of the human organism is still a subject of investigation [46,47,106]. The interplay between *M.tb* virulence factors and the host are in a constant battle whose outcome will determine whether the TB symptoms will manifest or whether the bacterium will be removed [107]. The identification of distinct immunological parameters would be good indicators for the performance of the vaccine pre, during or post-vaccination [106,107,108]. Knowledge of immune markers or the expression of *M.tb* virulence factors inside the host will give us a clear picture of the workings of the human organism during infection, vaccination, latency, and follow-up period [109], so that the treatment plan can be adjusted accordingly to provide maximum protection against TB.

### 4.2. Different M.tb Strains, Different Patterns

Different strains of *M.tb* respond to the same environment in a different way, but the mechanisms are yet to be explored in depth. When a drug or vaccine candidate is subjected to a *M.tb* challenge test, generally, “laboratory strains”, such as the CDC1551 strain or Erdman strain, are used. For example, the *M.tb* strain, Erdman, CDC1551 or Danish, or clinical virulent strains can significantly affect the endpoint and responses of study subjects [110,111]. The effectiveness or behavior of the tested candidates depends upon the type of strains used in the challenge study. Thus, it is difficult for researchers to correlate the results obtained from challenge with the CDC1551 strain or the Erdman strain (also known as “laboratory strains”) and compare them with clinical strains. Altogether, if such information is available to researchers, it will help them to design clinical trials and implementation strategies with precision [109,112,113,114].

### 4.3. Safety Concerns

One of the most important challenges in vaccine development is to maintain the safety of the recipient. For example, attenuated *M.tb* strains are more promising in providing maximum protection from resistant sensitive TB, but they cannot be used in the case of HIV–TB co-infection or in TB-endemic areas. This limits the use of attenuated *M.tb* strains for vaccination purposes. On the other hand, vaccines based on *M.tb* antigens (or subunit vaccine) induce specific immune responses and provide subdued protection; yet, they are relatively preferred because they are a safer option.

### 4.4. Lost in Transition

When laboratory-proven vaccine candidates are tested in clinical trials to study their effectiveness and readiness, they (mostly) fail. Researchers often initiate dialogue with other stakeholders, industry or public health experts quite late. For a single investigator or a group of investigators, it is difficult to perform or envision clinical trials, and by the time, they realize, in most the cases, a product is lost in the transition. A report published by Malcolm Macleod in 2018 [63] raised several important issues on conducting clinical trials, the correlation of animal studies, and regulatory guidelines. The MVA85A vaccine failed in the Phase b clinical trial, though satisfactory results were obtained in preclinical studies [61,62,63]. Macaque monkeys who were given MVA85A, exhibited adverse reactions compared to BCG control, and were euthanized post BCG vaccination [63]. Preclinical studies performed in the mice model and the primate model offered limited but crucial information with respect to the MVA85A vaccine, but these were not thoroughly considered while planning clinical trials [62,63]. In light of this evidence, it is recommended to consider the reproducibility of the experiments, the behavior and biological variability of animals, and the statistical parameters of preclinical studies. Thus, it is important for investigators to collaborate with fellow researchers, industry and public health experts, and medical practitioners; this diverse expertise will benefit the research. A program on “Clinical Trial Awareness” may be a good start.

### 4.5. Stages of Vaccine Administration According to Need

**Figure 2:**
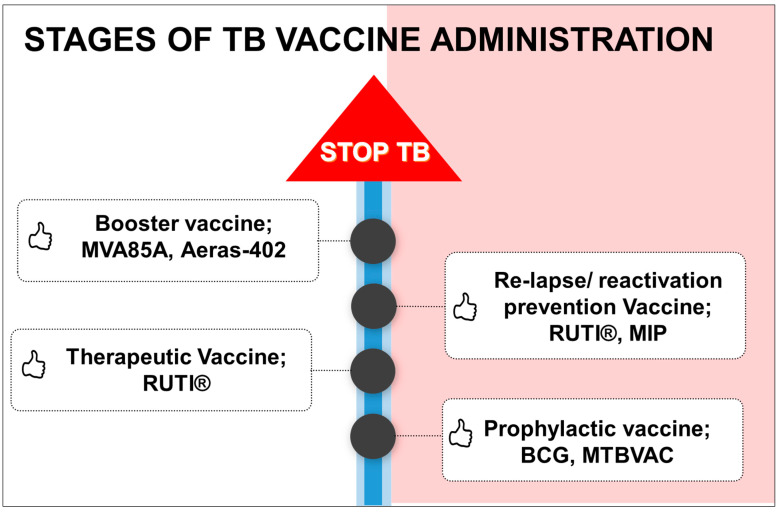
Stages of TB vaccine administration: TB has variability in disease manifestations; thus, there should be vaccine candidates that can be used specifically against TB symptoms rather than a general vaccine, i.e., BCG.

TB has a great deal of variability in disease manifestation; for example, pulmonary TB infection, extrapulmonary TB infection, latent TB infection (LTBI), relapse or re-activation. Hence, it is prudent to develop vaccine candidates that can be used specifically against TB rather than a general vaccine, i.e., BCG. Broadly, there may be four categories under which TB vaccine candidates can be developed, which are prophylactic, therapeutic, post-exposure, and booster (Figure 2). Figure 2 indicates that TB may not be controlled with a single prophylactic BCG vaccine but different vaccines are needed to control various stages of TB infection. Prophylactic vaccination is given to the non-infected individuals, which will prevent the infection at a later stage. BCG is a classic example of a prophylactic vaccine. Therapeutic vaccination is given to infected individuals exhibiting disease symptoms or undergoing treatment. A vaccine candidate, RUTI^®^, is being investigated for immunotherapeutic purposes. TB may re-occur in some of individuals even after the successful completion of TB treatment, suggesting that there is a lack of long-lasting memory T cells against *M.tb* epitopes, which can prevent the reactivation of TB. RUTI^®^ is in Phase II clinical trials to investigate its potential as a therapeutic vaccination in patients undergoing chemotherapy for drug-sensitive TB and drug-resistant TB infection [18]. Conceptually, a vaccine designed for preventing the re-activation of disease is likely to reduce the drug course and stimulate specific immune responses. An example is RUTI^®^ *M. indicus pranii*.

Finally, as the efficacy of the vaccine is weakened after a few years (or months) of vaccination, a booster dose is given to re-prime the vaccine and induce memory cells. The booster dose can also be given to bolster chemotherapy using an existing drug treatment [115,116]. The re-administration of the BCG vaccine as a booster (booster vaccination) may lead to adverse effects, especially in immunocompromised individuals, and hence it is better to develop alternative vaccine candidates for booster function [115,116]. Vaccine candidates such as MVA85A, Aeras-402 fall under this category.

### 4.6. Limitation in Using Appropriate Animal Models

Appropriate animal models are an important factor when evaluating the efficacy of novel vaccine candidates. Unfortunately, there is “no true animal reservoir” of *M.tb* that provides the most appropriate and detailed insights on TB pathology and can be used as a model testing the vaccine efficacy. Although close to 95% of the genes in humans and mice are similar, their antigen presentation function, and TB disease symptoms are different. In order to closely mimic the human immune response, a number of humanized mice models, e.g., HLA-A11, HLA-I or HLA-I/II have been developed as they can provide crucial breakthroughs in understanding TB pathogenesis, host–pathogen interaction, virulence factors of *M.tb*, as well as response to anti-TB drugs [91,117]. On the other hand, the human TB model is extremely complex and not available at the initial or proof-of-concept stage. Therefore, while performing preclinical studies using small animals (mice and rabbits) or non-human primates (monkeys), the utmost care should be taken for the selection of the correct model, experiment conditions, and the reproducibility of the results, as these drive the development of TB vaccine or anti-TB drugs to the next stage of human clinical trials.

## 5. Conclusions

In the last decade, several vaccine candidates have been identified, and there are approximately nineteen vaccine candidates in various phases of clinical trial: four in Phase I, eight in Phase II, six in Phase III, and one in Phase IV (Figure 1). Till today, no vaccine that prevents infection with drug resistant *M.tb* strain is available. This aspect needs special attention as the drug-resistant infection cases are increasing at an alarming rate [1]. The literature suggests that the use of attenuated or inactivated *M.tb* will protect to a broad spectrum of mycobacterial diseases. However, in the immunocompromised individuals, HIV patients, or in TB-endemic regions, the use of pathogenic *M.tb* strains in any form has greater risk of infection, re-infection or relapse cases. Thus, safer rBCG strains expressing one or a few mycobacterial proteins (fusion protein) as immunogens are considered suitable and safer vaccine candidates to use [10,115]. rBCG strains are being investigated for eliciting a wider range of CD4^+^/CD8^+^ T cell responses and for their longevity in protecting vaccinated individuals against multidrug-resistant TB [118]. Fusion protein/peptide antigens are weak immunogens and require the help of adjuvants to function. Thus, the development of protein/peptide antigens also depends upon the suitable adjuvants, which can improve the presentation of *M.tb* antigens and stimulate immune cells for innate and adaptive effector functions.

**Figure 3:**
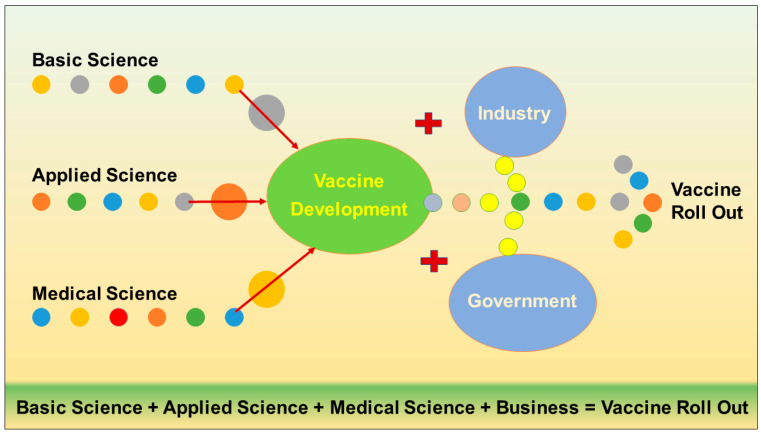
An overview of various factors and stakeholders across the globe involved in the vaccine development program.

As we have witnessed during the COVID-19 pandemic, accelerating the development of new and effective vaccines represents a crucial component of the global efforts to control the tuberculosis epidemic, including the spread of drug-resistant strains. For example, countries have spent nearly USD 90 billion on COVID-19 vaccine development in one year, whereas only USD 1.1 billion was invested in TB research and innovation in the last 11 years [119]. We have to extensively invest in collaborative research studies and conduct clinical trials in different parts of the world, with different ethnicities against different types of TB infections, i.e., pulmonary TB (pTB), extra-pulmonary TB (epPTB), and drug-resistant TB (Figure 3). Figure 3 suggests that in the vaccine development process, we need to consider “Collaboration, Cooperation, Consistency and Cost”. The management of TB should be prioritized from global scientific, administrative, and political think-tanks to achieve the ambitious target of “END TB” by 2030 [1].

## Figures and Tables

**Table 1:** Various types of vaccines designed against tuberculosis.

S. No	Vaccine Candidate	Antigen, Vector, and Formulation	Remark	References
Protein subunit vaccine				
1.	M72/AS01_E_	Fusion proteins containing *M.tb* 32A and *M.tb* 39A + adjuvant AS01E.	The vaccine was found to be clinically tolerated in *M.tb*-infected and *M.tb-*uninfected adults, and it is highly immunogenic. It induced multifunctional mycobacteria-specific T cell populations after vaccination, as well as boosted distinct T cells primed by natural *M.tb* infection. It provides protection against active pulmonary tuberculosis disease.	[4,8,9]
2.	H4:IC31	Fusion protein comprising *M.tb* Ag85B and TB10.4, formulated in IC31 adjuvant.	H4:IC31 was found to be an immunogenic and safe vaccine. In BCG-vaccinated adults, antigen-specific, long-lasting strong CD4^+^ T cells were observed.	[4,10]
3.	H56:IC31	Fusion protein comprising *M.tb* Ag85B, ESAT-6, Rv2660 formulated in IC31 adjuvant.	H56:IC31 is a safe vaccine. It induces antigen-specific IgG and Th1-type CD4^+^ T cells in healthy adults without or with *M.tb* infection. All recruited adults were HIV-negative and BCG-vaccinated.	[4,11]
4.	ID93-GLA	ID93 consists of *M.tb* antigens Rv2608, Rv3619c, Rv3620c, and Rv1813c formulated with GLA-SE adjuvant.	ID93 vaccine was studied in BCG-vaccinated, HIV-uninfected, drug-sensitive pulmonary tuberculosis patients. No adverse effect was observed. The vaccine was found to be immunogenic as it stimulated long-lasting, vaccine antigen-specific polyfunctional CD4^+^ T-cells and robust antibody production. Possible adjunctive therapeutic vaccine.	[12]
5.	GamTBvac	The vaccine contains Ag85A and ESAT-6:CFP-10 fusion plus adjuvant DEAE-dextran core and CpG oligodeoxynucleotides.	The vaccine was found to be safe and immunogenic, inducing Th1-type CD4^+^ T cells and IgG antibodies in BCG-vaccinated, *M.tb*-uninfected adults.	[13]
6.	AEC/BC02	The vaccine contains *M.tb* protein subunits Ag85B and ESAT-6: CFP-10 along with *Bacillus Calmette–Guérin* CpG plus aluminum adjuvant system.	AEC/BC02 enhanced the potential of chemotherapy for latent TB infection in mice model. The vaccine induced Th1-type immune responses and decreased bacillary load in latently infected mice.	[14]
Recombinant BCG (rBCG)				
7.	AFRO-1	AFRO-1 is a rBCG overexpressing *M.tb* antigens Ag85A, Ag85B, 10.4 and Perfringolysin O from *Clostridium* *perfringens*.	The vaccine was found to be safe and well-tolerated by immunocompromised SCID mice. Mice vaccinated with rBCG (AFRO-1) showed enhanced cellular immune response against *M.tb* (strain HN878) infection. AFRO-1-vaccinated mice had better survival rates than BCG-vaccinated mice challenged with *M.tb*. Enhanced immune response was also observed in guinea pigs.	[15]
8.	BCG- Revaccination	Whole-cell *M. bovis*.	(i) In BCG-vaccinated individuals, BCG-specific CD4^+^ T-cell responses were improved post BCG revaccination.	[16]
9.	BCG + H107	BCG plus H107 fusion protein (Rv3863, ESAT-6, EspI, EspC, EspA, MPT64, MPT70 and MPT83).	This vaccine stimulated the development of multifunctional T cell populations, induced Th17 response, and provided robust protection against pulmonary *M.tb* infection in mice.	[17]
10.	VPM1002	rBCG Prague strain overexpressing listeriolysin molecule from *Listeria* with inactivated urease subunit C.	VPM1002 vaccine is safe and has superior efficacy compared to BCG against *M.tb* infection in mice. The vaccine was found to be safe in various animal models, such as immune-deficient mice, guinea pigs, rabbits, and non-human primates. Phase I clinical trials have indicated safety.	[4,18]
Viral-mediated delivery of mycobacterial antigens				
11.	MVA85A	Vaccinia virus expressing *M.tb* antigen Ag85A.	MVA85A was found to be safe and well-tolerated in BCG-vaccinated adults. The vaccine induced robust and durable cellular immune responses when delivered intramuscularly or by aerosol route.	[19,20]
12.	AdHu5Ag85A	Replication-deficient adenoviral TB vaccine expressing *M.tb* Ag85A.	The vaccine-induced protection was observed against mycobacterial infections (*M. bovis, M.tb*) in animal models, such as mice, guinea pigs.	[21,22]
13.	AERAS-402	AERAS-402 is a replication-deficient Ad35 vaccine encoding the fusion protein of *M.tb* antigens 85A, 85B, and TB10.4.	The vaccine was safe, well-tolerated, and induced CD8^+^ T cell response in HIV-negative and BCG-vaccinated adults.	[23]
14.	TB/FLU-01L	Attenuated influenza strain Flu NS106 expressing *M.tb* antigen ESAT-6.	The vaccine was safe and showed immunotherapeutic effect in mice. The vaccine was found to be safe and immunogenic in BCG-vaccinated adults.	[24]
15.	TB/FLU-04L	Modified influenza vector with a truncated NS1 protein expressing *M.tb* antigens ESAT-6 and Ag85A.	TB/FLU-04L was found to be safe and induced protective immune response against *M.tb* infection in BCG-vaccinated adults.	[6]
16.	Vaccine regimen ChAdOx185A- MVA85A	Replication-deficient chimpanzee adenovirus vector expressing *M.tb* antigen 85A (Ag85A) + recombinant, replication-deficient modified vaccinia virus expressing *M.tb* antigen Ag85A.	Vaccine was safe in BCG-vaccinated adults.	[5]
17.	RhCMV/TB	A rhesus cytomegalovirus vector expressing 9 *M.tb* antigens such as Ag85A, Ag85B, ESAT-6 (acute phase), Rv3407, Rv1733, Rv2626 (latency), Rpf A, Rpf C, Rpf D (resuscitation).	The vaccine generated stronger, effector-differentiated CD4^+^ and CD8+ memory T cell responses, and induced protection against *M.tb* (Erdman strain) infection in rhesus macaques.	[25]
18.	rLCMV-based *M.tb* vaccine	Replication-deficient lymphocytic choriomeningitis virus (rLCMV) expressing *M.tb* antigens Ag85B and TB10.4.	The vaccine generated polyfunctional *M.tb*-specific CD4^+^ and CD8^+^ T cell populations in mice and reduced lung infection burden during challenge with *M.tb.*	[26]
*M.tb* mutants or inactivated or fragmented *M.tb* strains as vaccine candidates				
19.	MTBVAC	Live, attenuated *M.tb* clinical isolates—lineage 4 with deletion genes (*phoP* and *fadD26*).	MTBVAC is the only live attenuated vaccine, used in clinical trials as a preventive vaccine in newborns. MTBVAC is safe and immunogenic in BCG-vaccinated and HIV-negative adults.	[27]
20.	DAR-901	Inactivated whole-cell *M. obuense*.	DAR-901 was safe and tolerable in IGRA-negative, BCG-immunized adults in Tanzania. DAR-901 failed to prevent TB infection.	[4,28]
21.	IKEPLUS	*M. smegmatis* Δesx-3 locus (IKE) + *M.tb* esx-3 genes.	IKEPLUS induces protective immunity to *M.tb* in mice.	[29]
22.	*M. vaccae* based vaccine	Heat-killed non-tuberculous bacterium *M. vaccae.*	Improved bacterial clearance was observed in multidrug-resistant and drug-sensitive TB patients. It can prevent chemotherapy-induced hepatic damage and overcome weight loss and inflammation observed during TB infection.	[30]
23.	RUTI^®^	The vaccine contains fragmented, purified, and heat-inactivated *M.tb* bacilli in liposomes.	Reasonably safe and provides potent stimulation of the immune response against tuberculosis. RUTI^®^ showed polyfunctional T cell response and enhanced secretion of IFN-γ after vaccination, attempted to control MDR condition.	[31]
24.	*Mycobacterium indicus pranii* (MIP)	Inactivated whole-cell *M. indicus pranii*.	MIP was safe with no adverse effects. A role of MIP in clearance of the *M.tb* bacilli was also suggested.	[32]
25.	DNA-based Dual vaccine (pP24-*M.tb* DNA vaccine)	*M.tb* antigens, MPT64, Ag85A, Ag85B, and TB10.4 + a HIV protein, p24 as a backbone	pP24-*M.tb* vaccine was immunogenic, provided protection against *M. bovis* BCG, and reduced infection-related lung inflammation and injury in mice. Elicited robust immune responses to HIV-1. Promising vaccine to prevent dual infections with *M.tb* and HIV.	[33]

## Data Availability

Not applicable.
